# Evaluating the effect of mutations and ligand binding on transthyretin homotetramer dynamics

**DOI:** 10.1371/journal.pone.0181019

**Published:** 2017-07-13

**Authors:** Tadeo. E. Saldaño, Giuseppe Zanotti, Gustavo Parisi, Sebastian Fernandez-Alberti

**Affiliations:** 1 Universidad Nacional de Quilmes/CONICET, Bernal, Argentina; 2 Department of Biomedical Science, University of Padua, Padova, Italy; Universidade Nova de Lisboa Instituto de Tecnologia Quimica e Biologica, PORTUGAL

## Abstract

Native transthyretin (TTR) homotetramer dissociation is the first step of the fibrils formation process in amyloid disease. A large number of specific point mutations that destabilize TTR quaternary structure have shown pro-amyloidogenic effects. Besides, several compounds have been proposed as drugs in the therapy of TTR amyloidosis due to their TTR tetramer binding affinities, and therefore, contribution to its integrity. In the present paper we have explored key positions sustaining TTR tetramer dynamical stability. We have identified positions whose mutations alter the most the TTR tetramer equilibrium dynamics based on normal mode analysis and their response to local perturbations. We have found that these positions are mostly localized at β-strands E and F and EF-loop. The monomer-monomer interface is pointed out as one of the most vulnerable regions to mutations that lead to significant changes in the TTR-tetramer equilibrium dynamics and, therefore, induces TTR amyloidosis. Besides, we have found that mutations on residues localized at the dimer-dimer interface and/or at the T4 hormone binding site destabilize the tetramer more than the average. Finally, we were able to compare several compounds according to their effect on vibrations associated to the ligand binding. Our ligand comparison is discussed and analyzed in terms of parameters and measurements associated to TTR-ligand binding affinities and the stabilization of its native state.

## Introduction

Amyloid diseases involve protein misfoldings into cross-β-sheet structures and aggregations, ultimately leading to amyloid fibril formation. Amyloid fibrils deposition on various tissues causes systematic organ dysfunctions like cardiomyopathy, ophthalmopathy, and neuropathy. While most of current treatments are limited to reduce the symptoms, numerous clinical trials deal with other alternative therapies. Among them, transthyretin(TTR) results as a possible molecular target of amyloidosis disruption. TTR misfolds and misassembles are responsible of hereditary amyloidosis like senile systemic amyloidosis[1], familial amyloid polyneuropathy[2], and familial amyloid cardiomyopathy[3].

TTR is an amyloidogenic protein whose native homotetramer is involved in the transport of thyroxine hormone (T4) in plasma. The four TTR monomers are assembled with D2 symmetry. Each monomer is composed of two four-stranded antiparallel β-sheets and a short α-helix. Two monomers form a dimer stabilized by a hydrogen bond network between the two β-strands H and F at the monomer-monomer interface. Two of such dimers form the tetramer with a weaker dimer-dimer interface stabilized mostly by hydrophobic contacts between pairs of AB and GH loops. Two T4 hormone binding sites are located in the long hydrophobic cavities formed by each symmetric half of the dimer-dimer interface.

TTR tetramer dissociation into dimers is a required early step in the amyloid cascade[4],[5],[6]. The resulted pair of dimers quickly dissociates into monomers and, as a consequence, partial monomer denaturation leads to fibril assembly and other aggregates formation. It is therefore expected that TTR tetramer stabilization contributes to inhibit TTR fibrillogenesis[7],[8],[9],[10],[11],[12],[13],[14],[15],[16].

Specific point mutations that destabilize the TTR tetramer increase the propensity of fibril formation[9],[16],[17],[18]. The understanding at the molecular level of TTR tetramer equilibrium dynamics requires identification of the network of residues whose mutations directly affect their relative structural and dynamics stability.

The binding of thyroxine hormone T4 inhibits TTR amyloidogenesis by establishing interactions with residues at the dimer-dimer interface that bridge both subunits. Several drugs that bind to the hydrophobic T4 binding site are known to stabilize the native TTR tetramer conformation, and therefore slow amyloidogenesis[19],[20],[21]. The exploration of new classes of compounds with certain affinity to the TTR tetramer enlighten about promising candidates in the therapy against the disease.

Here in, we present a systematic selection of key positions sustaining TTR tetramer stability. Adapting a previously developed method[22] to identify key position residues whose mutations directly affect the affinity for a ligand, we identify key position residues whose mutations significantly alter the TTR tetramer equilibrium dynamics. The method makes use of normal mode analysis and mode responses to local perturbations. This combination has been previously proved as a useful tool to simulate effect of mutations in a large variety of proteins and, therefore, identify functionally important residues[22].

TTR tetramer ligand-free and ligand-bound conformations co-exist as local minima within the TTR energy landscape. The conformational changes involved in ligand-binding should be achieved by its intramolecular vibrational dynamics. We explore the possibility that the relatively high affinity of certain ligand to TTR can be a consequence of significant changes introduced on the subspace of normal modes of the tetramer involved in the unbound-to-bound conformational changes. If ligand binding modifies these modes, energy barriers that dissociate the TTR-ligand complex will be harder to overcome by thermal fluctuations. In a previous paper[22] we have presented a procedure to define and compare normal mode subspaces associated to ligand-binding. In the present paper we adapt this method to analyze TTR-ligand complex dynamics stability. In order to contribute with new complementary dynamics features to future explorations of potential new drugs, we compare several ligands according to their effect on vibrations associated to ligand binding.

## Methods

### Normal modes analysis

Normal mode analysis has been calculated using the coarse-grained Elastic Network Model (ENM)[23],[24],[25],[26],[27]. Normal modes and molecular dynamics, with the help of powerful computational tools that allow further structural and dynamics analysis[28],[29], become standard techniques for analyzing and enhancing our understanding of molecular mechanisms. Briefly, ENM represents the *N* residues of a protein by their α-carbons (nodes), connected by uniform springs to their neighbours within a cut-off distance *r*_*c*_. Here in, a value of *r*_*c*_ = 7Å has been used for all the selected TTR structures.

The potential of the network of *N* nodes is defined as[23],[30], [31]

$$E\left(r_{i},r_{j}\right)=\frac{1}{2}k_{ij}{\left(\left|r_{ij}\right|-\left|r_{ij}^{0}\right|\right)}^{2}$$ (1)

where ***r***_*ij*_ ≡ ***r***_*i*_−***r***_*j*_ is the vector connecting nodes *i* and *j*, and the zero superscript indicates the equilibrium position that corresponds to the coordinates of the α-carbons in an experimental structure. The type of interaction between nodes *i* and *j* defines the value of the force constant *k*_*ij*_ as[32]:

$$\begin{array}{c} \text{if}\;\left|i-j\right|=1\;\Rightarrow \;k_{ij}=\gamma \\ \text{else}\\ \text{if}\;\left|r_{ij}^{0}\right|\leq r_{c}\;\text{then}\\ \text{if}\;i\;\text{and}\;j\;\text{are connected by disulphide bridge}\;\Rightarrow \;k_{ij}=\gamma \\ \text{if}\;i\;\text{and}\;j\;\text{interact by hydrogen bond or salt bridge}\;\Rightarrow \;k_{ij}=\gamma \text{ x }0.1\\ \text{otherwise}\;\Rightarrow \;k_{ij}=\gamma \text{ x }0.01\\ \text{if}\;\left|r_{ij}^{0}\right|\geq r_{c}\;\Rightarrow k_{ij}=0 \end{array}.$$ (2)

being *γ* a scaling constant to match the theoretical B-factors with the experimental ones. RING program[33],[34] has been used to define inter-residue connectivities.

Normal modes are obtained by diagonalizing the 3*N*x3*N* Hessian matrix **H, a** matrix of second-order partial derivatives of the potential energy

$$\Lambda \;=\;q^{T}Hq$$ (3)

where **q** is an orthogonal matrix whose columns **q**_k_ are the eigenvectors of **H**, that is, the normal modes, and **Λ** is the diagonal matrix of eigenvalues λ_k_ of **H**.

### A. Mode perturbations, comparison and key residues

Single point mutations of a residue *i* are simulated by introducing perturbations to the local interactions involving the *i*^th^ residue[35], [36], [37], [38]. Previous works have shown that this can be performed by changing the force constants *k*_*ij*_ that connect *i* with other residues *j* by a small amount δγ = 0.05 [22]. Here in, in order to simulate mutations introduced in the TTR tetramer, we simultaneously modify the force constants of the four *i*^th^ residue on each monomer. The new set of perturbed normal modes ${\left\{\;q_{k}^{i}\right\}}_{k=1,4\text{x}3N-6}$ (*N* being the number of residues of each monomer) is compared with the unperturbed {**q**_*k*_}_*k* = 1,2x3*N*−6_ modes as follows.

Firstly, we calculate the overlap matrix **O** with elements defined as the dot product

$${\text{O}}_{kk^{\prime }}^{i,}=q_{k}^{i}\cdot \;q_{k^{\prime }}^{\;}$$ (4)

The similarity R^*i*^is defined as

$${\text{R}}^{i}=\frac{\sum_{k=1}^{4\text{x}3N}{\left({\text{O}}_{k}^{i,max}\right)}^{1/2}}{4\text{x}3N}$$ (5)

with ${\text{O}}_{k}^{i,max}$ defined as the maximum value of ${\left({\text{O}}_{kk^{\prime }}^{i}\right)}^{2}$ among the projections ${\text{O}}_{kk^{\prime }}^{i}$ (*k*′ = 1, 4x3*N*) of $\;q_{k}^{i}$ mode on the {**q**_*k*_}_*k* = 1,4x3*N*_ modes.

The lower the value of R^*i*^, the larger effects that mutations in the residue *i* introduce on the TTR tetramer equilibrium dynamics. The effects of mutations of residue *i* on the TTR tetramer equilibrium dynamics are finally evaluated by normalizing R^*i*^ as

$${\text{Z}}^{i}=\frac{{\text{R}}^{i}-\bar{R}}{\sigma^{\text{R}}}$$ (6)

Where $\bar{R}$ and *σ*^R^ are the average and standard deviation of the distribution of R^*i*^ over all residues. Key position residues are selected as those presenting relative large negative values of Z^*i*^. That is, residues with large negative values of Z^*i*^ are residues whose mutations alter the most the TTR tetramer equilibrium dynamics.

### B. Subspace of modes associated to ligand-binding: Definition and comparison

Dynamics associated to tetramer-ligand assembly is defined as the subspace of normal modes that overlap the most with the direction dictated by structural distortions introduced by ligand binding. The procedure to select these modes follows the procedure previously described elsewhere[22]. Firstly, unbounded TTR tetramer and TTR tetramer-ligand complex structures are superposed minimizing the root-mean-square-deviation (RMSD) between Cα atoms. The normalized difference vector **v** between them is projected on the basis of the ligand-free tetramer normal modes

$$v=\sum_{k=1}^{4\text{x}3N-6}\left(v\cdot \;q_{k}^{\;}\right)\;q_{k}^{\;}=\sum_{k=1}^{4\text{x}3N-6}\left(\sum_{j=1}^{4\text{x}3N}\left(v_{j}q_{jk}^{}\right)\right)\;q_{k}^{\;}=\sum_{k=1}^{4\text{x}3N-6}c_{k}\;q_{k}^{\;}$$ (7)

with

$$c_{k}=\sum_{j=1}^{4\text{x}3N}\left(v_{j}q_{jk}^{}\right)$$ (8)

We calculate the mode participation number[39],[40],[22] as a quantitative measure of the degree of delocalization of **v** among the different tetramer normal modes

$$P_{q}={\left(\sum_{k=1}^{3N-6}{\left(c_{k}\right)}^{4}\right)}^{-1}$$ (9)

The value of P_***q***_, rounded to the nearest higher integer, indicates the size of the subspace of modes that retains the direction of the ligand-binding. Values of P_***q***_ ≈ 4x3*N*−6 mean that all vibrations of the tetramer are involved in the process, while values of P_***q***_ ≈ 1 indicate that the direction of structural changes is dictated by one single normal mode. The first P_***q***_ modes ordered by index *f*_*k*_ in decreasing values of (*c*_*k*_)^2^ define the subspace **S** of modes ${\left\{q_{f_{i}}\right\}}_{i=1,P_{q}}$ required to achieve a good description of the conformational change. In this way, **S** retains normal modes most involved in the tetramer-ligand complex formation.

Once defined **S**, the corresponding subspace **S**^ligand^ is obtained by establishing a one-to-one correspondence between ligand-free tetramer’s normal modes {**q**_*k*_}_k = 1,4x3*N*−6_ and normal modes ${\left\{\;q_{k}^{\text{ligand}}\right\}}_{k=1,4\text{x}3N-6}$ calculated using the tetramer-ligand complex structure. This is achieved by maximizing of the trace of the square of the overlap matrix **O** whose elements are defined as the dot product

$${\text{O}}_{kk^{\prime }}^{}=q_{k}\cdot q_{k^{\prime }}^{\text{ligand}}$$ (10)

This can be done by using a variant of the Min-Cost algorithm[41],[42] that selects those elements of the **O** matrix, one for each row, and each pertaining to a different column (or vice versa), which maximize the sum of their squared values.

The comparison of ligand-free tetramer and ligand-bound tetramer subspaces of modes, **S** and **S**^ligand^, associated to the conformational change upon ligand-binding is performed through the calculation of the corresponding Gramian matrix[43],[44], [45], [46], [22]. Firstly, each $q_{j}^{\text{ligand}}$ is projected onto the set of modes ${\left\{\;q_{k}^{\;}\right\}}_{k=P_{q}}$ and the corresponding vector projection is obtained as

$$p_{j}^{S^{\text{ligand}}S}=\sum_{k=1}^{P_{q}}\left(q_{j}^{\text{ligand}}\cdot q_{k}\right)q_{k}$$ (11)

The Gramian matrix **G** of the set of vectors ${\left\{p_{j}^{S^{\text{ligand}}S}\right\}}_{j=P_{q}}$, being P_***q***_ the dimension of subspaces, is calculated as the matrix of inner products with elements

$${\text{G}}_{kl}=\left(p_{k}^{S^{\text{ligand}}S}\cdot p_{l}^{S^{\text{ligand}}S}\right)$$ (12)

By diagonalizing **G**

$$L_{G}^{T}GL_{G}=\Lambda_{G}$$ (13)

we obtain eigenvalues ${\left\{\lambda_{k}\right\}}_{k=1,P_{q}}$ that vary in the [0:1] range. Therefore, the similarity of the two subspaces can be quantify as

$$\zeta^{S^{\text{ligand}}S}=\frac{\sum_{k}^{M}\lambda_{k}}{P_{q}}$$ (14)

The smaller the value of $\zeta^{S^{\text{ligand}}S}$, the stronger the effect of the ligand on tetramer vibrations associated to the conformational changes required in the TTR tetramer-ligand complex dissociation. As a consequence, TTR tetramer-ligand complex dissociation slows down and the complex gain stability.

$\zeta^{S^{\text{ligand}}S}$ depends on subspace dimensionalities. Therefore, for each TTR tetramer-ligand complex in the dataset we normalize the values of $\zeta^{S^{\text{ligand}}S}$ as:

$$Z_{\text{score}}^{S^{\text{ligand}}S}=\frac{\zeta^{S^{\text{ligand}}S}-\bar{\zeta^{ref}}}{\sigma^{ref}}$$ (15)

where $\bar{\zeta^{ref}}$ and *σ*^*ref*^ are the average and standard deviation of a reference distribution built by performing the comparison of **S**^ligand^ with subspaces **S**^ref^ obtained by randomly selection of *P*_***q***_ ligand-free normal modes within windows of ± 10 modes centered on each original **q**_*k*_ of subspace **S**.

## Results and discussion

**Fig 1:**
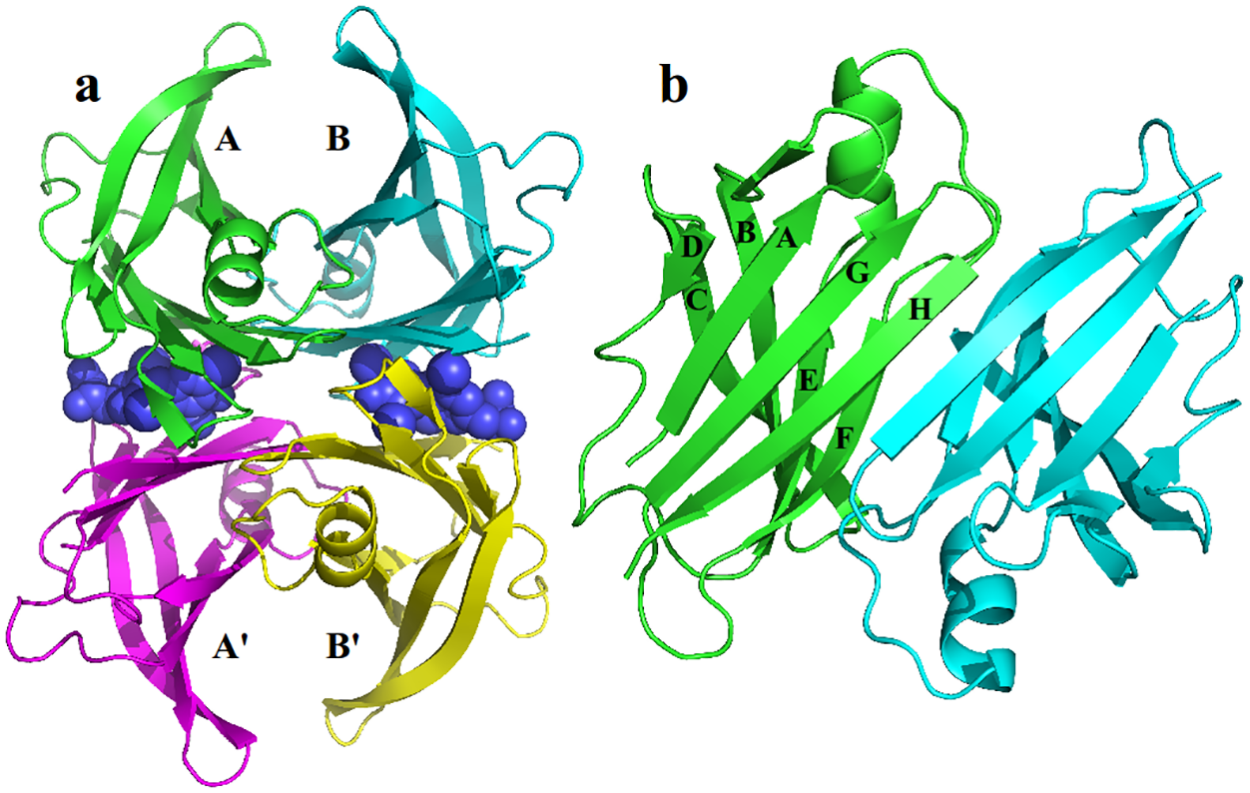
Structure of TTR-T4 complex. T4 molecules are displayed in the binding cavities as spheres and TTR monomers as ribbons. (a) The two monomers present in the asymmetric unit of the crystal structures used are labelled as A and B, symmetry related monomers as A’ and B’. (b) View of the A-A’ monomer-monomer interface.

The structure of the tetrameric TTR-T4 complex is depicted in Fig 1. T4 hormones are localized at the hydrophobic cavities of each dimer-dimer interface. Dimers are composed of two monomers (A-A’ and B-B’ respectively). Each monomer is formed by two four antiparallel β-strands and a short α-helix. The monomer-monomer interfaces are stabilized by hydrogen bond networks between the two β-strands H and F generating eight-stranded β-sandwich tertiary structures. The list of structures of TTR tetramer-ligand complexes used in this work is indicated in Table 1, including ligand free TTR tetramer. We applied several filters to TTR structures available in the PDB database in order to obtain a well curated dataset: (i) crystal structures with resolution < 1.7 Å, (ii) structures without missing residues between reside 11 and 124, (iii) structures without mutations, (iv) crystal structures with optimal Spearman rank correlation coefficient between experimental and theoretical B-factors > 0.6 Å, (v) structures with ligands that have been demonstrated to inhibit fiber formation, (vi) structures with ligands whose relative affinities have been proved experimentally using either competition binding assays of isothermal titriation calorimetry (ITC).

**Table 1:** TTR PDB coordinates used in this paper.

TTR-ligand complex	PDB code
Ligand-free	1f4l[47]
T4	2rox[48]
T4-resveratrol	5cr1[49]
Tafamidis	3tct[11]
Diflunisal	4i89[13]
TBBPA	5hjg[50]
Apigenin	4wo0[51]
CHF5074	4i85[13]
Daidzein	5al8[49]
Quercetin	4wnj[51]
Pterostilbene	4wns[51]
Genistein	5akv[49]
Resveratrol-3-O-sulfate	5al0[49]

### Effect of mutations on dynamics

The intramolecular vibrational dynamics of a protein is intrinsically related to its structural stability. Therefore, changes in the TTR tetramer equilibrium dynamics can be associated to changes in its relative stability. It is expected that mutations with large effects on TTR tetramer dynamics will destabilize it, increasing the propensity of TTR fibril formation and amyloid diseases.

Single point mutations have been simulated on each residue of the ligand-free TTR tetramer structure in order to identify key position residues sustaining TTR tetramer dynamics stability. The value of Z^*i*^ (see Section Mode perturbations, comparison and key residues) is a measure of the change in the TTR tetramer equilibrium dynamics introduced by mutations on the *i*^th^ residue. The lower the value of Z^*i*^, the larger the effect of mutations of residue *i*^th^ on the tetramer stability.

**Fig 2:**
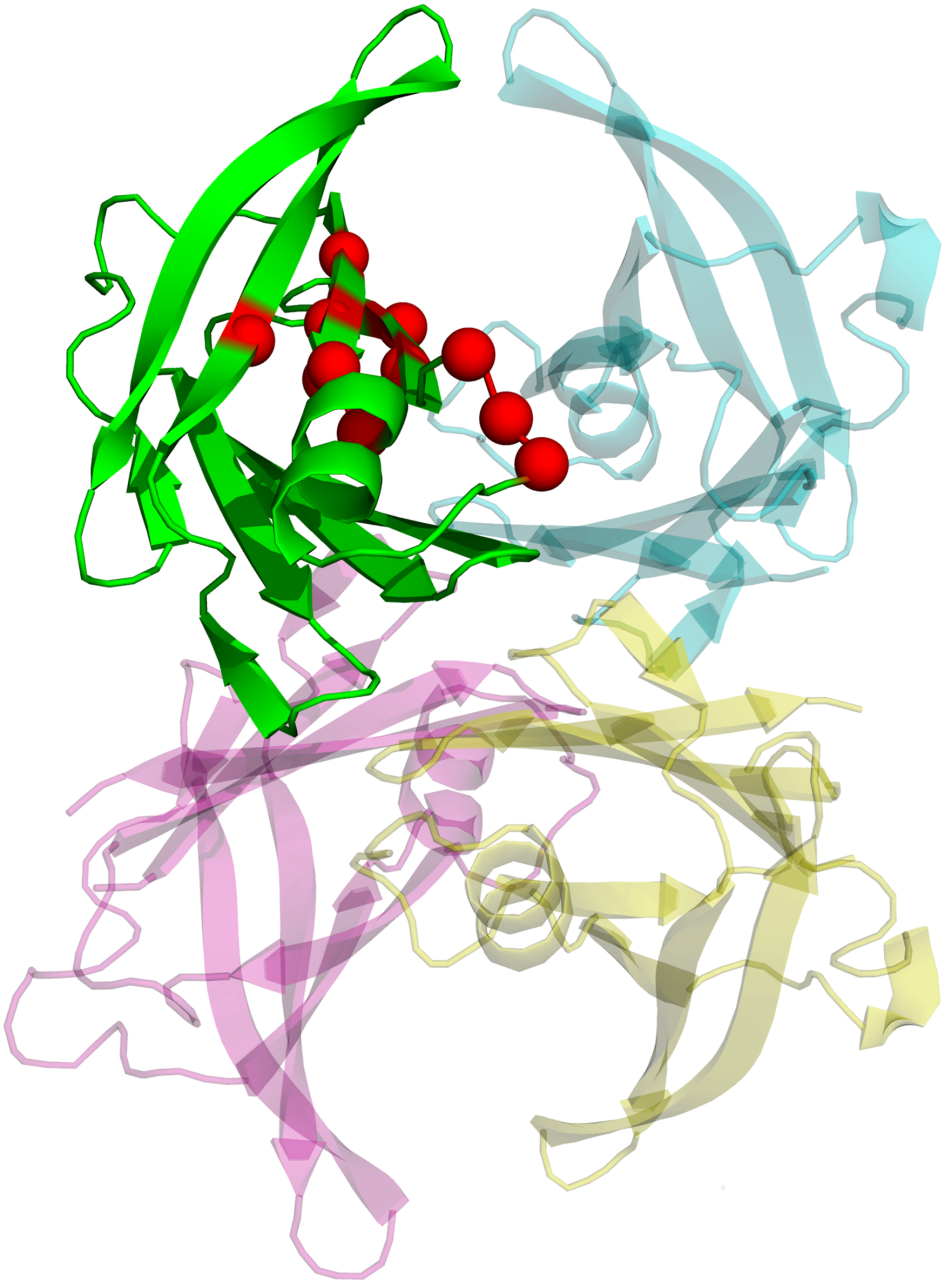
Key position residues shown as spheres. Their localizations are displayed in only one monomer.

Firstly, in Table 2 we analyse key position residues selected as those ranked with the lowest 10% values of Z^*i*^. Most of them are localized in the loop segment β-strands E, EF-loop, and β-strands F. For the sake of robustness, it is important to mention that results shown in Table 2 were mostly reproduced using two ligand-free TTR tetramer structures availables (PDB_id: 1f4l and 2qgb). The RMSD between these structures is 0.47Å. Despite changes in the ordering, similar qualitative results have been obtained for both structures. More than 75% of residues ranked with the lowest 20% values of Z^*i*^ are reproduced on both structures and ~90% match by +/- one residue. The complete list of the lowest 20% values of Z^*i*^ obtained using both ligand-free structures is provided in S1 and S2 Tables. Fig 2 shows their localization within the TTR structure. Almost half of them are located at the monomer-monomer interface, revealing as one of the most vulnerable regions to mutations that lead to significant changes in the TTR-tetramer equilibrium dynamics. Interestingly, in Table 2 we also show that among top 10% positions with low Z^*i*^, most of them correlates with mutations previously reported to induce TTR amyloidogenesis. Particularly, V30, with four different mutations in this position and with Z^*i*^ = −1.17, has been identified as one of the most prominent familial amyloid polyneuropathy mutation[14],[16],[52].

**Table 2:** Key position residues.

Residue	*Z^i^*	Secondary Structure Element	Type	Intra- monomer IACs	Inter-monomer IACs	Mutation	<ΔΔG>
His 88	-1,87	EF-loop	*	7	1	His → Arg[56]	-1,03
Tyr 69	-1,58	β-strand E		13		Tyr → His; Tyr → Ile	18.03
Thr 75	-1,52	α-helix		9			8,18
Phe 95	-1,46	β-strand F	*	9	2		20,56
Phe 87	-1,44	EF-loop	*	4	6	Phe → Met	18,53
Ile 73	-1,38	β-strand E		11		Ile → Val	17,49
Glu 89	-1,33	EF-loop	*	5	3	Glu → Gln; Glu → Lys	3,55
Val 71	-1,32	β-strand E		9		Val → Ala	16,81
Val 93	-1,22	β-strand F	*	6	2	Val → Met	9,74
Val 30	-1,17	β-strand B		7		Val → Leu; Val → Met Val → Ala; Val → Gly	23,46
Asp 74	-1,15	β-strand E		7		Asp → His (Non Amyloidogenic)	7,63

**Type**:* monomer-monomer interface[47]

**IAC**: *Inter Atomic Contact*.

**Mutations**: http://www.amyloidosismutations.com/mut-attr.php

Key position residues have been analyzed using using FoldX algorithm[53],[54] that predicts differences in the free energy changes ΔΔG = ΔG_mutant_-ΔG_wild-type_ between the unfolding free energy of a mutant (= ΔG_mutant_) and wild type (ΔG_wild-type_) protein. The average values ΔΔG over all possible mutation performed on each key position residue are listed in Table 2. In good agreement with our predictions, we found that most key position residues are strongly destabilizing (ΔΔG>2kcal/mol)[55].

**Fig 3:**
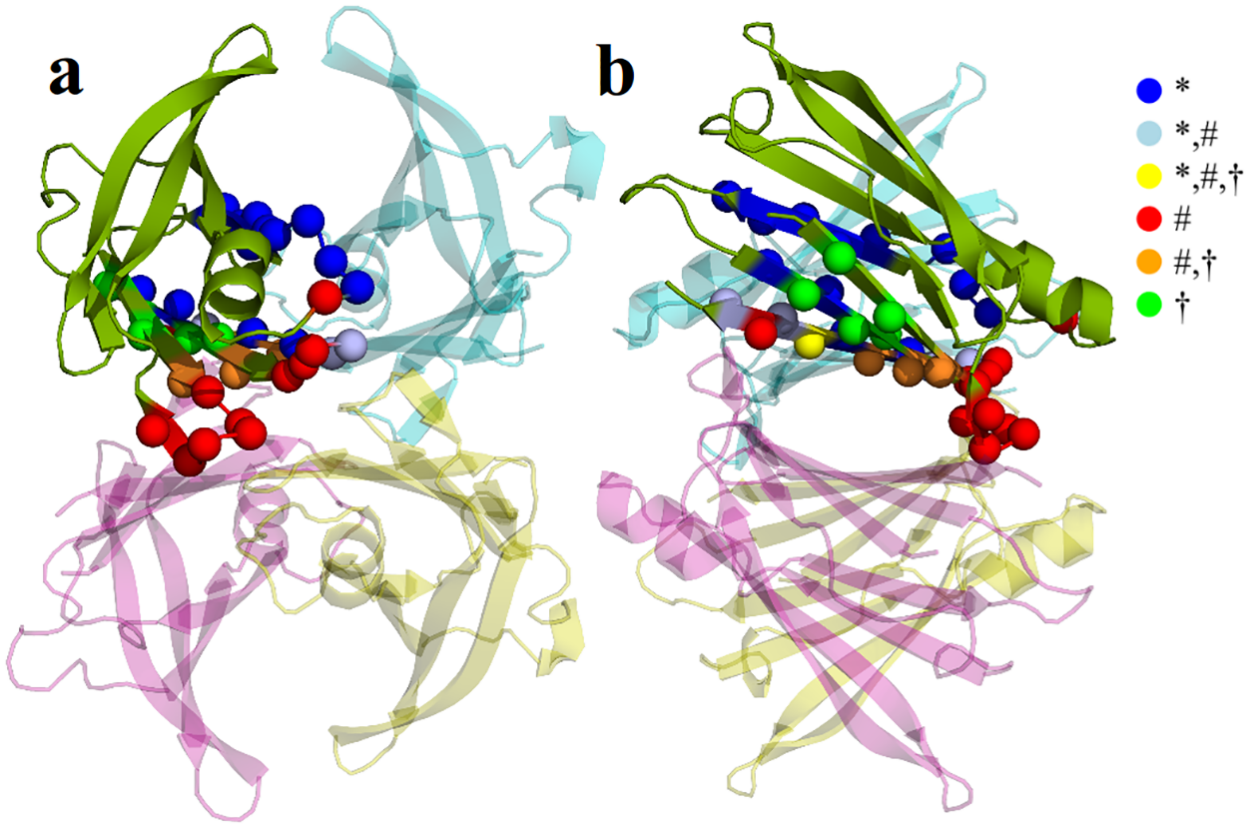
Residues of substrate-binding cavity, monomer-monomer, and dimer-dimer interfaces shown as spheres. Their localizations are displayed only in one monomer. (* monomer-monomer interface, **#** dimer-dimer interface, **†** substrate-binding cavity).

Table 3 shows values of Z^*i*^ for residues previously identified as belonging to the thyroxine hormone binding site (TBS), monomer-monomer, and dimer-dimer interfaces [47], [48], [57], [58]. All of the substrate-binding cavity residues present negative values of Z^*i*^, indicating that mutations on these residues destabilize the tetramer more than the average. T4 binding sites are at the weakly dimer-dimer interfaces. We observe negative values of Z^*i*^ in dimer-dimer interface residues within the large L110-V122 fragment (β-strands G, GH-loop, and β-strands H), and L17-D18 (β-strands A), while positive values are observed for A19-S23 segment (AB-loop) and S85 (EF-loop). Finally, all monomer-monomer interface residues also present negative values of Z^*i*^. That is, mutations on any of the residues of the large F87-V122 segment introduce larger perturbations on the tetramer structure than the average. Fig 3 shows the localization of all these residues within the TTR structure.

**Table 3:** Z^i^ values of residues of substrate-binding cavity, monomer-monomer, and dimer-dimer interfaces.

Residue	Z^*i*^	Secondary Structure Element	type	Intra-monomer IACs	Inter-monomer IACs	Mutation
Met 13	-0,16	β-strand A	**†**	9		Met → Ile (Non Amyloidogenic)
Lys 15	-0,45	β-strand A	**†**	8		
Leu 17	-0,21	β-strand A	**#,†**	8	2	
Asp 18	-0,30	β-strand A	**#**	8	1	Asp → Asn; Asp → Gly; Asp → Glu
Ala 19	0,15	AB-loop	**#**	7	3	Ala → Asp
Val 20	0,18	AB-loop	**#**	6	2	Val → Ile
Arg 21	0,53	AB-loop	**#**	6	1	
Gly 22	0,19	AB-loop	**#**	4	1	
Ser 23	0,14	AB-loop	**#**	5	1	Ser → Asn
Ser 85	0,31	EF-loop	**#**	4	1	
Phe 87	-1,44	EF-loop	*	4	6	
His 88	-1,87	EF-loop	*	7	1	
Glu 89	-1,33	EF-loop	*	5	3	Glu → Gln; Glu → Lys
His 90	-0,92	EF-loop	*	5	1	His → Asp; His → Asn (Non Amyloidogenic)
Glu 92	-0,53	β-strand F	*	6	4	Gln → Lys
Val 93	-1,22	β-strand F	*	6	2	Val → Met
Val 94	-1,08	β-strand F	*	6	4	Val → Ala
Phe 95	-1,46	β-strand F	*	9	2	
Thr 96	-0,93	β-strand F	*	4	3	
Tyr 105	-1,04	β-strand G	*	14	1	
Thr 106	-0,37	β-strand G	**†**	4		
Ile 107	-1,01	β-strand G	*	9	1	Ile → Val; Ile → Phe Ile → Met
Ala 108	-0,43	β-strand G	**†**	4		
Ala 109	-0,61	β-strand G	**†**	6		Ala → Ser (Non Amyloidogenic) Ala → Thr (Non Amyloidogenic)
Leu 110	-0,06	β-strand G	**#,†**	6	2	
Ser 112	-0,21	β-strand G	**#**	7	1	Ser → Ile
Pro 113	-0,62	GH-loop	**#**	9	1	
Tyr 114	-1,07	GH-loop	*,**#**	6	6	Tyr → His; Tyr → Cys
Ser 115	-0,18	β-strand H	*,**#**	5	4	
Tyr 116	-0,70	β-strand H	*	6	6	Tyr → Ser
Ser 117	0,44	β-strand H	**#,†**	3	2	
Thr 118	-0,33	β-strand H	*	5	1	
Thr 119	-0,58	β-strand H	*, **#, †**	3	5	Thr → Met (Non Amyloidogenic)
Ala 120	-0,91	β-strand H	*, **#**	4	2	Ala → Ser
Val 121	-0,54	β-strand H	**#**	4	2	
Val 122	-0,40	Loop	*, **#**	5	3	Val → Ile; Val → del; Val → Ala

**Type**: * monomer-monomer interface[47]

**#** dimer-dimer interface [47]

**†** substrate-binding cavity [48], [50], [57].

**IAC**: *Inter Atomic Contact*.

**Mutations**: obtained from: http://www.amyloidosismutations.com/mut-attr.php

### A. Effect of ligand-binding on dynamics

**Fig 4:**
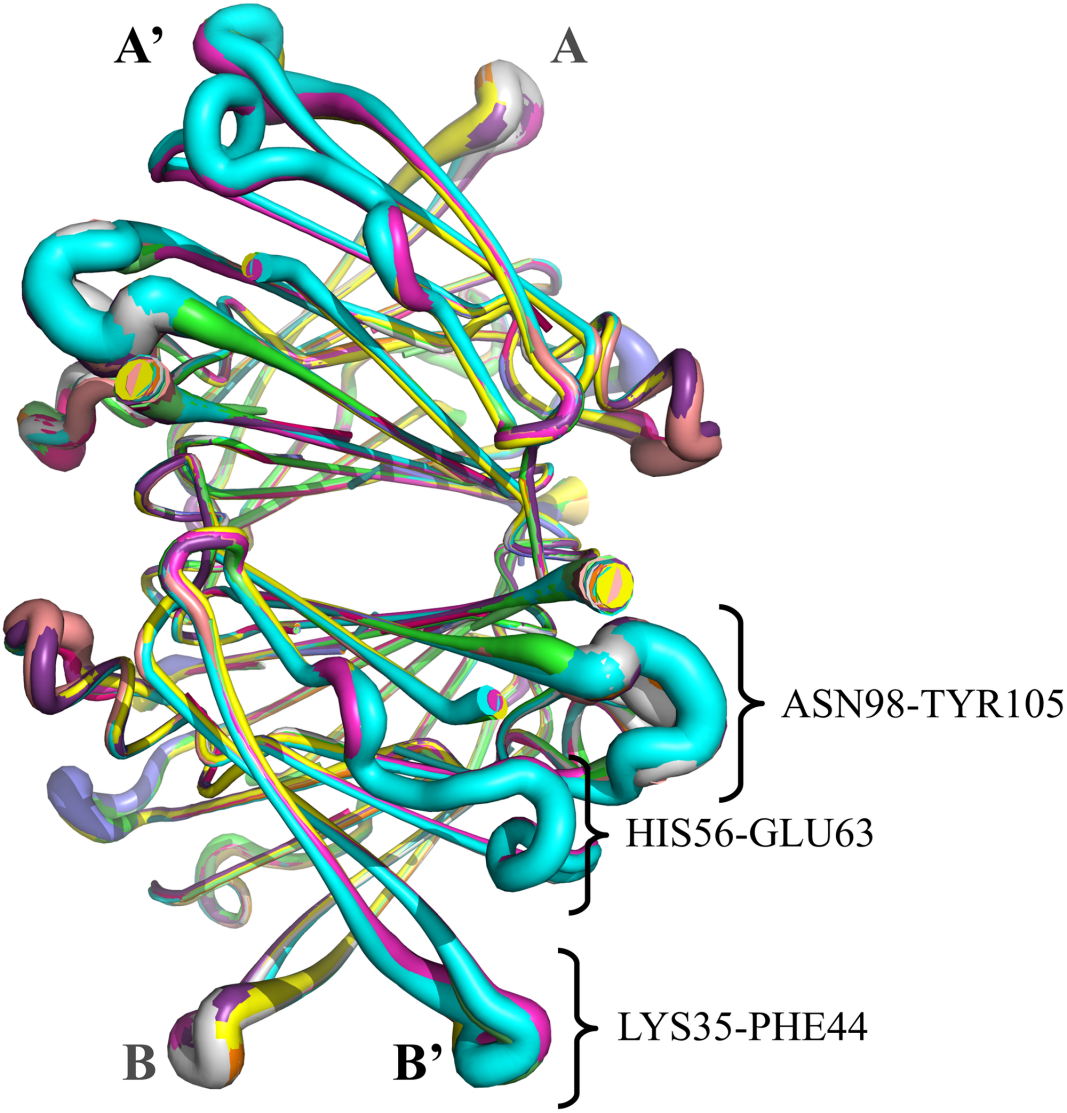
Superimposed putty-style cartoon representations of the 13 structures TTR tetramer-ligand complexes listed in Table 1. The variable-width corresponds to values of experimental B-factors. The segments of sequence with larger flexibilities are also indicated.

**Fig 5:**
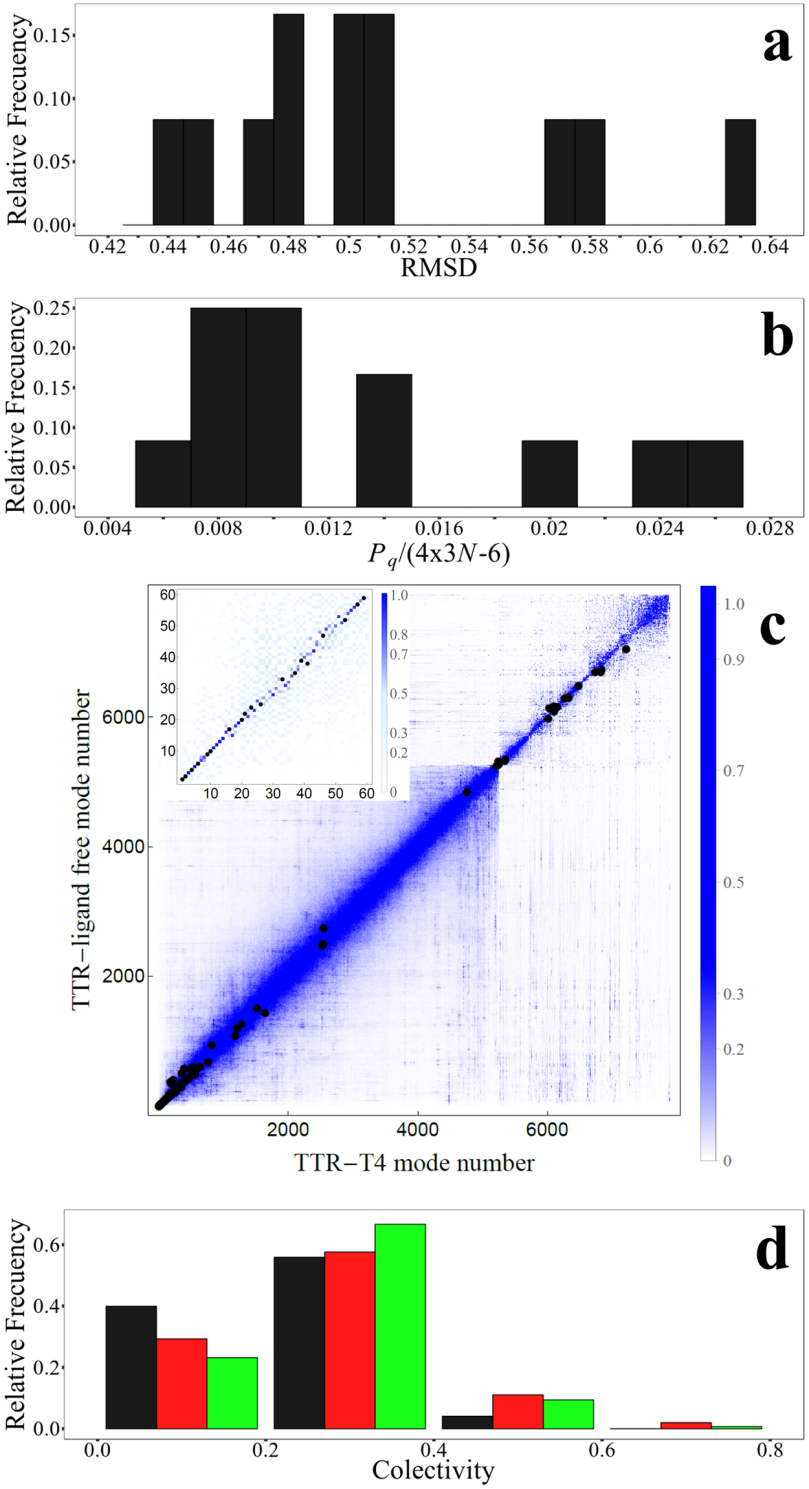
(a) Distribution of RMSD between TTR tetramer ligand-free structure and each TTR tetramer-ligand complex structure; (b) Distribution of the fraction of normal modes involved in the ligand-induced conformational transition calculated as *P*_***q***_/4x3*N*; (c) overlap matrix between TTR tetramer ligand-free and TTR-T4 complex normal modes highlighting modes belonging to the ligand-binding subspace (black dots), the inset displays the overlap matrix for modes within the low-frequency range; (d) Distribution of degree of collectivity, κ_k_, for each normal mode that participates in the conformational change (red), for each normal mode that participate significantly in the flexibility pattern (green), and for all other modes (black).

We have performed a comparative analysis of effects of several drugs and compounds (see Table 1) on TTR tetramer ligand-free vibrations involved in the conformational changes associated to TTR tetramer-ligand complex formation/dissociation. Fig 4 shows the superimposed structures listed in Table 1 indicating those regions presenting larger flexibilities. None of the key position residues listed in Table 2 belong to one of these regions. The structural distortions introduced by ligand-binding are analyzed in terms of the RMSD (root mean square difference) between TTR tetramer ligand-free structure and each TTR tetramer-ligand complex structure. Fig 5A displays the corresponding distribution of RMSD values. The low values of RMSD indicate that TTR tetramer ligand-free structure is not largely affected by ligand-bindings. The largest value of 0.63 corresponds to the RMSD with TTR tetramer-T4 complex structure. A detailed information of RMSD values between all structures of our dataset is shown in S1 Fig. TTR tetramer-T4 (pdb: 2ROX) complex structure is the structure that presents the main structural differences with the rest of the structures, mainly due to structural differences between T4 and the other compounds. Despite that, RMSD values between TTR tetramer complexes seem not to follow a clear relation with chemical similarities between ligands, mostly belonging to the large family of polyphenols.

Furthermore, Fig 5B shows the distribution of the fraction of TTR tetramer ligand-free normal modes involved in ligand-induced conformational transitions calculated as values *P*_***q***_/(4x3*N*−6) obtained over all TTR tetramer-ligand complexes in our dataset. The average value of *P*_***q***_ = 104 **±** 54, and *P*_***q***_/(4x3*N*−6) = 0.01± 0.007 indicates that a significantly small fraction of the vibrational space participates in the process of ligand-binding. In order to analyze the composition of subspace of normal modes associated to ligand-binding, Fig 5C depicts the overlap matrix between TTR tetramer ligand-free and TTR-T4 complex normal modes, highlighting modes belonging to the ligand-binding subspace. As it can be seen, normal modes associated to ligand-binding are not uniformly distributed throughout the whole range of modes. Mostly of these modes are localized at the low-frequency range and only a minor contribution corresponds to high frequency modes. This is in good agreement with previous studies that associate low-frequency normal modes with ligand-binding conformational changes[59],[60].

Normal modes can be analyzed in terms of their collectivities, defined as[59]

$$\kappa_{k}=\frac{1}{N}\text{exp}\left(-\sum_{i=1}^{N}{\left(q_{i,k}^{r}\right)}^{2}\text{ln}{\left(q_{i,k}^{r}\right)}^{2}\right)$$ (16)

being ${\left(q_{i,k}^{r}\right)}^{2}=\;{\left(q_{i,k}^{x}\right)}^{2}+{\left(q_{i,k}^{y}\right)}^{2}+{\left(q_{i,k}^{z}\right)}^{2}$, and ${\left(q_{i,k}^{j}\right)}^{2}$ (*j* = *x*, *y*, *z*) the components of the *i*th C_α_ residue in the *k* normal mode. *κ*_*k*_ = *N*^−1^ corresponds to normal modes equally distributed throughout all the residues of the protein, and *κ*_*k*_ = 1 represents normal modes localized on a single residue. Fig 5D points out that modes involved in ligand-induced conformational transition are composed of vibrations within the entire range of frequencies, showing a distribution of collectivities slightly higher than other modes. Besides, normal modes that participate of ligand-binding can be compared with essential normal modes involved in the TTR-tetramer flexibility pattern. In order to do that, vector **B**^lf^ with elements ${\text{B}}_{\text{i}}^{\text{lf}}$ corresponding to the B-factors associated to each i^th^ residue is expanded on the basis of {**q**_*k*_}_*k* = 1,4x3*N*−6_ modes

$$B^{\text{lf}}=\sum_{k=1}^{3N-6}\left(B^{\text{lf}}\cdot q_{k}\right)q_{k}=\sum_{k=1}^{3N-6}\left(\sum_{j=1}^{3N}\left(B_{j}^{\text{lf}}q_{jk}\right)\right)q_{k}=\sum_{k=1}^{3N-6}b_{k}q_{k}$$ (17)

with

$$b_{k}=\sum_{j=1}^{3N}\left(B_{j}^{\text{lf}}q_{jk}\right)$$ (18)

The mode participation number *P*_**B**_ associated to this expansion can be defined as

$$P_{B}={\left(\sum_{k=1}^{3N-6}{\left(b_{k}\right)}^{4}\right)}^{-1}$$ (19)

with an equivalent interpretation as *P*_**q**_ described in **Section Subspace of modes associated to ligand-binding: definition and comparison**. The first *P*_**B**_ modes ordered by index *f*_*k*_ in decreasing values of (*b*_*k*_)^2^ represent the minimum set of modes ${\left\{q_{f_{i}}\right\}}_{i=1,P_{B}}$ required to achieve a good representation of the flexibility pattern. The distribution of collectivities of modes related to the flexibility pattern of TTR-tetramer is also displayed in Fig 5D. We can see that subspaces of normal modes associated to ligand-binding do not involve the most collective modes that significantly contribute to protein flexibility.

**Fig 6:**
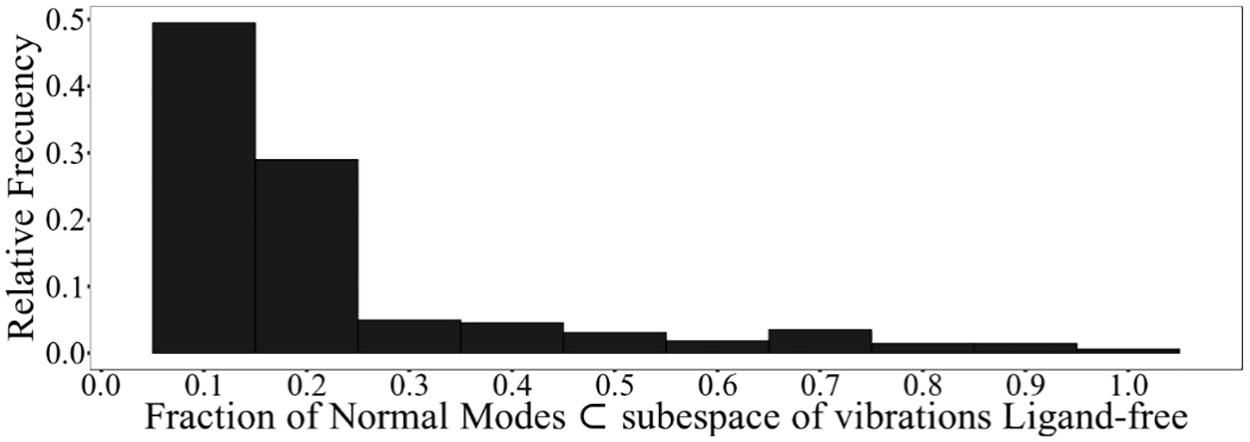
Distribution of fraction of normal modes shared between each ligand-binding subspace and T4-binding subspace.

**Fig 7:**
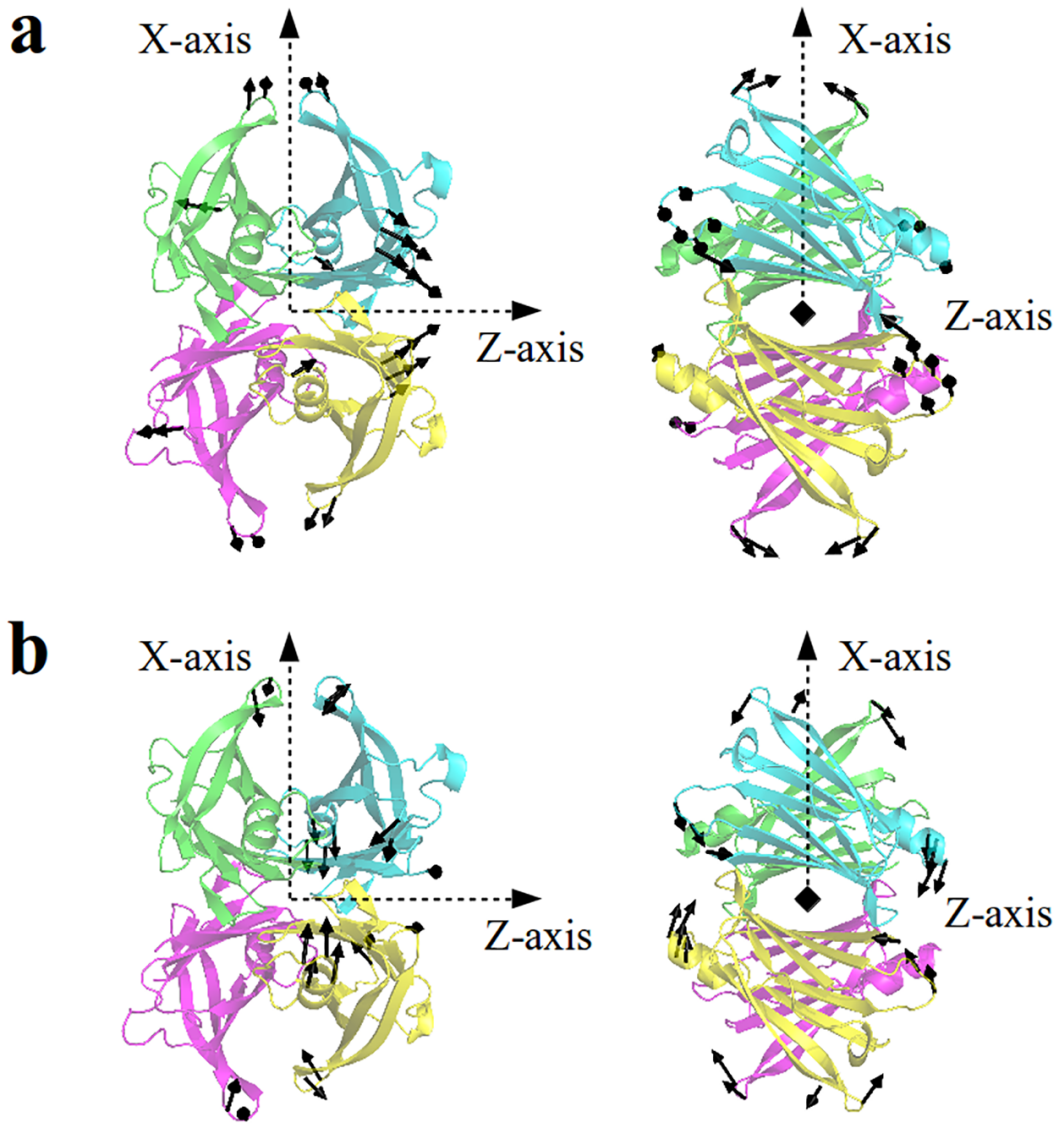
Normal modes 22(a) and 25(b) involved in conformational changes of all ligand-binding of our dataset.

In order to identify common dynamics aspects, we compare ligand-binding subspaces using the subspace for T4-binding as template. Fig 6 shows the distribution of fraction of modes shared between each ligand-binding subspace and T4-binding subspace. While most normal modes change among ligands, two normal modes (normal modes # 22 and 25) are shared by all ligand-binding. Fig 7 depicts these modes. Both modes present 2-fold rotational symmetry with respect to Z axis. They imply a combination of shear (mode #22) and hinge (mode # 25) motions between dimers on only one of the T4-binding cavities. Both modes imply relative motions mainly localized on EF-loop, FG-loop and BC-loop affecting only one of both dimer-dimer interfaces. Previous studies[51] show that TTR ligands present different abilities to saturate the two T4 binding sites, leading to the presence of a main binding site with clear ligand affinity and a second minor site with a much lower ligand affinity. Our results complement these previous structural evidence for asymmetric TTR-ligand binding related to its negative binding cooperativity[61], [62].

**Fig 8:**
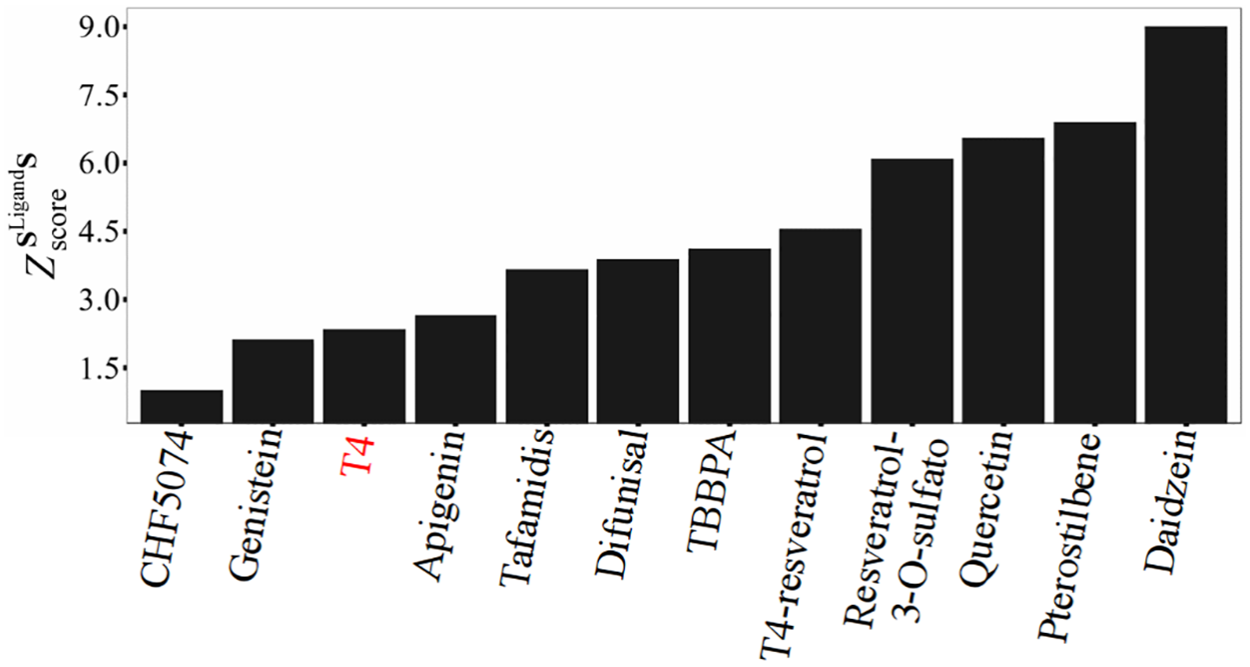
Values of $Z_{\text{score}}^{S^{\text{ligand}}S}$ for different ligands.

In Fig 8 we display the comparison of our set of ligands and compounds according to their effect on vibrations associated to ligand binding. In order to relate binding affinities to changes introduced on vibrations, we analyze our relative values of $Z_{\text{score}}^{S^{\text{ligand}}S}$ in terms of experimental parameters and measurements associated to TTR-ligand binding affinities and the stabilization of its native state. Ligands presenting low values of $Z_{\text{score}}^{S^{\text{ligand}}S}$ (see **Section Subspace of modes associated to ligand-binding: definition and comparison**) introduce significant changes on tetramer vibrations associated to the conformational changes required in the TTR tetramer-ligand complex dissociation. As a consequence, TTR tetramer-ligand complex gain stability and fibrillogenesis is inhibited.

Performing competition binding assays, it has been shown that the polyphenols pterostilbene and quercetin have different preferential binding sites in TTR than the one of T4[51]. Besides, experiments reveal that, at equal concentrations, quercetin is able to displace pterostilbene[51], in agreement with the lower value of $Z_{\text{score}}^{S^{\text{ligand}}S}$ for quercetin with respect to pterostilbene.

While neither pterostilbene nor quercetin is able to displace TTR-bond T4, the polyphenol apigenin displaces it but only at very high concentrations[51]. These results indicate that apigenin presents lower affinity than T4. This is in agreement with our higher value of $Z_{\text{score}}^{S^{\text{ligand}}S}$ for apigenin respect to T4. Even more, tafamidis TTR-affinity has shown to be higher than polyphenols and T4, something that $Z_{\text{score}}^{S^{\text{ligand}}S}$ can only confirm in the case of pterostilbene and quercetin. Nevertheless, apigenin, T4, and tafamidis are close in order of $Z_{\text{score}}^{S^{\text{ligand}}S}$.

According to competition experiments using resveratrol and radiolabeled T4 as probes, the resveratrol is not able to displace the bound T4. Besides, fluorescence experiments shows that resveratrol metabolites, like resveratrol-3-O-sulfate, have lower affinities for TTR than that of resveratrol[51]. In good agreement with that, we obtain $Z_{\text{score}}^{S^{\text{ligand}}S}$ (T4)< $Z_{\text{score}}^{S^{\text{ligand}}S}$(resveratrol)< $Z_{\text{score}}^{S^{\text{ligand}}S}$ (resveratrol-3-O-sulfate). Furthermore, fluorometric competitive binding assays, using resveratrol as fluorescent probe, indicates that CHF5074 presents high binding affinity for TTR, in agreement with its low $Z_{\text{score}}^{S^{\text{ligand}}S}$ value.

TTR-ligand dissociation constants (K_d_) determined by isothermal titriation calorimetry (ITC)[63] indicates that K_d_(diflunisal) = 580 nM is higher than K_d_(apigenin) = 250 nM, in agreement with values of $Z_{\text{score}}^{S^{\text{ligand}}S}\left(\text{apigenin}\right)<Z_{\text{score}}^{S^{\text{ligand}}S}\left(\text{diflunisal}\right)$. Furthermore, ITC experiments also report values of K_d_(TBBPA) = 20 nM and K_d_(Tafamidis) = 3 nM[50], leading to a final order of affinity to TTR as tafamidis>TBBPA>diflunisal. Values of $Z_{\text{score}}^{S^{\text{ligand}}S}\left(\text{TBBPA}\right)>Z_{\text{score}}^{S^{\text{ligand}}S}\;\left(\text{diflunisal}\right)$ do not agree with these results, despite both compounds are close in order of $Z_{\text{score}}^{S^{\text{ligand}}S}$.

Relative capacities of genistein, apigenin and daidzen to displace TTR-bound radiolabeled T4 indicates the order of binding affinities for TTR as genistein>apigenin>daidzen, in good agreement with the order given in Fig 8. Among them, only genistein and apigenin are able to displace TTR-bound resveratrol while daidzen results a weak ligand exhibiting rather low binding affinity. That is, TTR-binding affinities varies as genistein> apigenin> resveratrol> daidzen in agreement with the order $Z_{\text{score}}^{S^{\text{ligand}}S}\left(\text{genistein}\right)<Z_{\text{score}}^{S^{\text{ligand}}S}\left(\text{apigenin}\right)<Z_{\text{score}}^{S^{\text{ligand}}S}\left(\text{resveratrol}\right)<Z_{\text{score}}^{S^{\text{ligand}}S}\left(\text{daidzen}\right)$.

At this point it is interesting to mention that values of $Z_{\text{score}}^{S^{\text{ligand}}S}$ do not correlate neither with values of RMSD between ligand-free and ligand-bound structures nor with values of ΔIACs(Inter Atomic Contacts) changes. We obtain Spearman correlation coefficients of -0.22 between $Z_{\text{score}}^{S^{\text{ligand}}S}$ and RMSD and -0.07 between $Z_{\text{score}}^{S^{\text{ligand}}S}$ and ΔIACs. Therefore, $Z_{\text{score}}^{S^{\text{ligand}}S}$ do not depend on these structural features. Ligands that introduce larger structural distortions or changes of inter-residue interactions do not correspond to those that modify the most the TTR-tetramer equilibrium dynamics.

## Conclusions

We have analyzed the effects of mutations and ligand-binding pattern on TTR-tetramer equilibrium dynamics. We have explored key positions residues that most affect TTR-tetramer vibrations. Most of them are localized at β-strands E, EF-loop, and β-strands F, with amyloidogenic mutations previously reported. The monomer-monomer interface is pointed out as one of region most vulnerable to mutations that lead to significant changes in the TTR-tetramer equilibrium dynamics and, therefore, induces TTR amyloidogenesis. Besides, we have found that mutations on residues localized at the dimer-dimer interface and/or at the T4 hormone binding site destabilize the tetramer more than the average.

At this point it is important to mention that, in the present work, we explore the effects of mutations on equilibrium dynamics of TTR tetramer. It is expected that this particular aspect, among others structural features, is associated to the stability of the quaternary structure. Considering the diversity of reasons that lead a mutation to be pro-amyloidogenic, a correlation between reported amyloidogenic TTR mutations and Z^*i*^, that only quantifies perturbations of TTR tetramer equilibrium dynamics, is not expected. Nevertheless, values of Z^*i*^ not only represent a complementary contribution to explore new potential mutations but also enlighten about effects of some of the previously reported mutations.

Several drugs and compounds have been compared according to their effect on vibrations associated to the ligand binding. We found that a significantly small fraction of the vibrational space participates of the ligand-induced conformational transitions. We discussed these particular ligand effects in terms of experimental parameters and measurements associated to TTR-ligand binding affinities and stabilization of its native state. In general, the effect of ligands on equilibrium dynamics of ligand-free TTR-tetramer is in good agreement with previous findings obtained with competition binding assays.

Two normal modes have been found to participate in all ligand-bindings of our dataset. Both of them imply motions between dimers on only one of the T4-binding cavities, evidencing the asymmetric TTR-ligand binding related to its negative binding cooperativity.

As resume, in the present paper we focus on the analysis of effects of mutations and ligand-binding on TTR-tetramer equilibrium dynamics. We have found that our analysis of dynamics aspects leads to complementary information that can help for future developments of new drugs against amyloid diseases.

## Supporting information

S1 Fig RMSD values between all structures of our dataset. (TIF)

S1 Table Residues with the lowest 20% values of Z^*i*^ obtained using ligand-free structure PDB_id: 1f4l. (PDF)

S2 Table Residues with the lowest 20% values of Z^*i*^ obtained using ligand-free structure PDB_id: 2qgb. (PDF)
