# Supplementary figures and images for: Evaluating the effect of mutations and ligand binding on transthyretin homotetramer dynamics
Source: PLoS One. 2017 Jul 13;12(7):e0181019. doi: 10.1371/journal.pone.0181019 (PMC5509292; doi:10.1371/journal.pone.0181019)

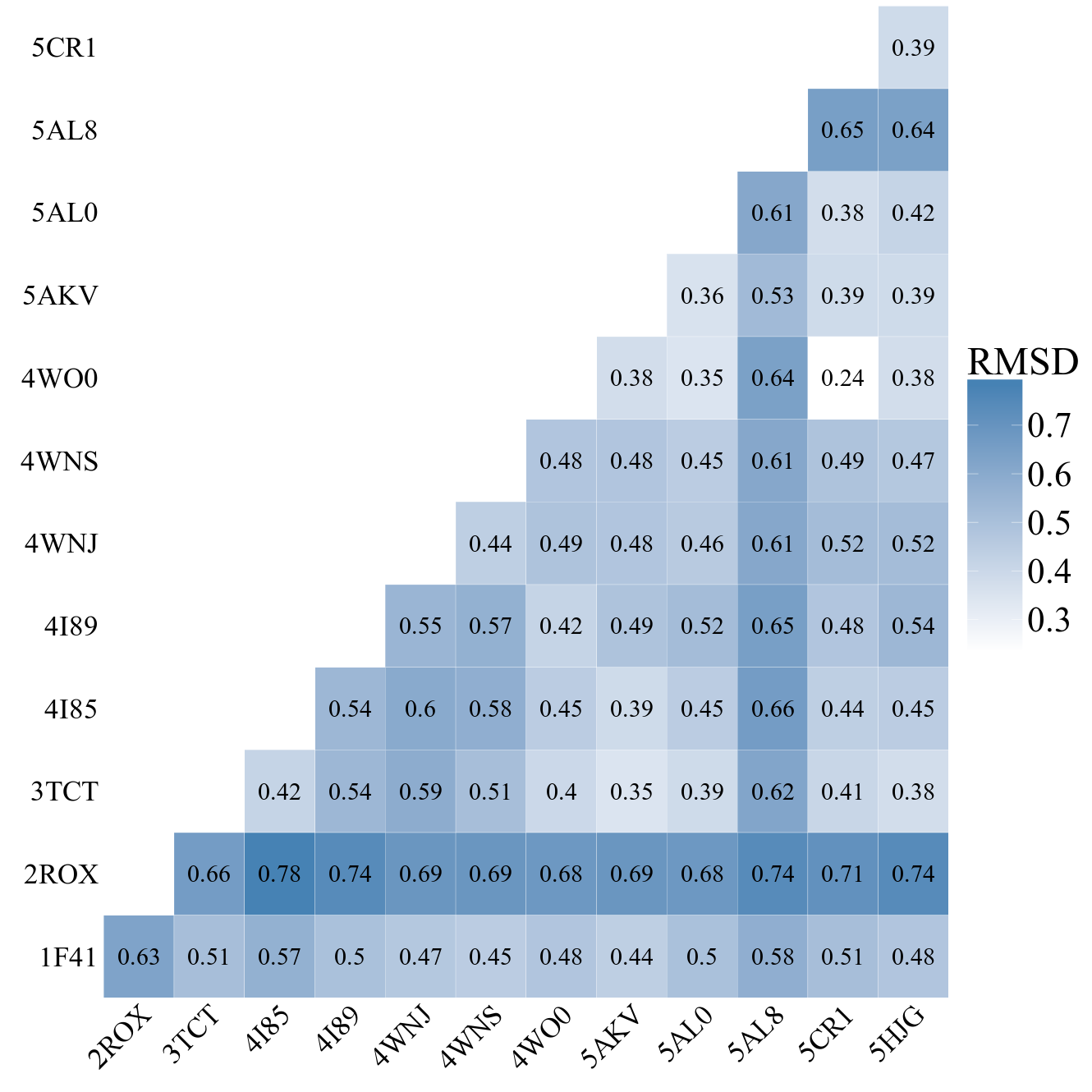

Supplement: S1 Fig — (TIF) [file pone.0181019.s001.tif]
