# Supplementary material for: Evaluating the effect of mutations and ligand binding on transthyretin homotetramer dynamics
Source: PLoS One. 2017 Jul 13;12(7):e0181019. doi: 10.1371/journal.pone.0181019 (PMC5509292; doi:10.1371/journal.pone.0181019)
Supplement: S1 Table — (PDF) [file pone.0181019.s002.pdf]

Table S1. Key position residues – pdb: 1f41

Residue	Z^i	Secondary Structure Element	Type	Intra-monomer IACs	Inter-monomer IACs	Mutation
His 88	-1,87	EF-loop	*	7	1	His → Arg[56]
Tyr 69	-1,58	β-strand E		13		Tyr → His; Tyr → Ile
Thr 75	-1,52	α-helix		9		
Phe 95	-1,46	β-strand F	*	9	2	
Phe 87	-1,44	EF-loop	*	4	6	Phe → Met
Ile 73	-1,38	β-strand E		11		Ile → Val
Glu 89	-1,33	EF-loop	*	5	3	Glu → Gln; Glu → Lys
Val 71	-1,32	β-strand E		9		Val → Ala
Val 93	-1,22	β-strand F	*	6	2	Val → Met
Val 30	-1,17	β-strand B		7		Val → Leu; Val → Met<br>Val → Ala; Val → Gly
Asp 74	-1,15	β-strand E		7		Asp → His<br>(Non Amyloidogenic)
Glu 72	-1,11	β-strand E		6		Glu → Gly
Leu 111	-1,10	β-strand G		11		Leu → Met
Lys 70	-1,09	β-strand E		7	1	Lys → Asn
Val 94	-1,08	β-strand F	*	6	4	Val → Ala
Tyr 114	-1,07	GH-loop	*,#	6	6	Tyr → His;Tyr → Cys<br>Tyr → Cys
Tyr 105	-1,04	β-strand G	*	14	1	
Ile 107	-1,01	β-strand G	*	9	1	Ile → Val;Ile → Phe<br>Ile → Met
Tyr 78	-0,99	α-helix		12		Tyr → Phe
Val 28	-0,98	β-strand B		9		Val → Met
Ala 91	-0,98	β-strand F		5		Ala → Ser
Thr 96	-0,93	β-strand F	*	4	3	

Type:* monomer-monomer interface[47]
# dimer-dimer interface [47]
† substrate-binding cavity [48],[50], [57].
IAC: *Inter Atomic Contact.*
Mutations*: http://www.amyloidosismutations.com/mut-attr.php*
