# Supplementary material for: Evaluating the effect of mutations and ligand binding on transthyretin homotetramer dynamics
Source: PLoS One. 2017 Jul 13;12(7):e0181019. doi: 10.1371/journal.pone.0181019 (PMC5509292; doi:10.1371/journal.pone.0181019)
Supplement: S2 Table — (PDF) [file pone.0181019.s003.pdf]

Table S2. Key position residues – pdb: 2rox

Residue	Z^i	Secondary Structure Element	Type	Intra-monomer IACs	Inter-monomer IACs	Mutation
Tyr 105	-1,6	β-strand G	*	14	1	
Phe 87	-1,54	EF-loop	*	4	6	Phe → Met
Thr 75	-1,49	α-helix		9		
His 88	-1,45	EF-loop	*	7	1	His → Arg[56]
Tyr 69	-1.33	β-strand E		13		Tyr → His; Tyr → Ile
Ile 73	-1,24	β-strand E		11		Ile → Val
Pro 86	-1,23	EF-loop		7		
Phe 95	-1,19	β-strand F	*	9	2	
Thr 120	-1,16	β-strand H	*,#	4	2	Ala → Ser
Val 93	-1,15	β-strand F	*	6	2	Val → Met
Trp 79	-1,1	α-helix		13		
Ile 107	-1,08	β-strand G	*	9	1	Ile → Val;Ile → Phe<br>Ile → Met
Asp 74	-1,02	β-strand E		7		Asp → His<br>(Non Amyloidogenic)
Val 71	-0.97	β-strand E		9		Val → Ala
Val 14	-0,93	β-strand A		8		
Thr 96	-0,92	β-strand F	*	4	3	
Glu 89	-0,9	EF-loop	*	5	3	Glu → Gln; Glu → Lys
Val 121	-0,89	β-strand H	#	4	2	
Leu 111	-0,88	β-strand G		11		Leu → Met
Val 94	-0,87	β-strand F	*	6	4	Val → Ala
Tyr 114	-0,86	GH-loop	*,#	6	6	Tyr → His;Tyr → Cys<br>Tyr → Cys
Lys 76	-0,84	α-helix		8	1	

Type:* monomer-monomer interface[47]
# dimer-dimer interface [47]
† substrate-binding cavity [48],[50], [57].
IAC: *Inter Atomic Contact.*
Mutations*: http://www.amyloidosismutations.com/mut-attr.php*
